# Neuroprotective effects of glycine against cisplatin-induced cognitive decline in mice

**DOI:** 10.1097/MS9.0000000000004955

**Published:** 2026-05-01

**Authors:** Hafiz Muhammad Obaid Kazmi, Najeeb Ullah, Muhammad Ikram, Iqra Khadam, Umer Khalil, Syeda Iqra Maqsood, Saima Mumtaz Khattak, Muhammad Umer Suleman, Muhammad Mustafeez Waheed Jami, Muhammad Mursaleen, Shahbaz Azam Khan, Sumaira Javed, Syeda Nafisa Tabassum

**Affiliations:** a Department of Anatomy, Ayub Medical College, Abbottabad, Pakistan; b Institute of Basic Medical Sciences, Khyber Medical University, Peshawar, Pakistan; c Institute of Pharmaceutical Sciences, Khyber Medical University, Peshawar, Pakistan; d Jehlum College of Nursing and Health Sciences, Pakistan; e Department of Obstetrics & Gynaecology, Ayub Teaching Hospital, Abbottabad, Pakistan; f Federal Medical College, Islamabad, Pakistan; g DHQ (WM&DC) Abbottabad, Pakistan; h Department of Microbiology, Bangladesh Rural Advancement Committee, Dhaka, Bangladesh

**Keywords:** antioxidant, cisplatin, cognitive dysfunction, inflammation, neuroprotection, neurotoxicity syndromes, oxidative stress

## Abstract

**Background:**

Cisplatin is a common chemotherapeutic agent known to cause impairments often referred to as “chemo-brain.” Preclinical evidence suggests cisplatin induces toxicity via oxidative stress and neuroinflammation.

**Methods:**

Twenty-five healthy adult male BALB/c mice (*n* =5 per group) were randomly assigned to five treatment cohorts over a 28-day period. Cisplatin (3 mg/kg) was administered intraperitoneally every fourth day, and glycine (1 g/kg/day) was administered subcutaneously daily. Groups assessed concurrent protection and delayed/reversal intervention. Spatial working memory (Y-maze/spontaneous alternation percentage) and spatial learning [Morris water maze (MWM)/latency to platform] were evaluated before and after treatment using paired T-tests and one-way analysis of variance (ANOVA).

**Results:**

Cisplatin administration alone resulted in significant cognitive decline in the Y-naze (*P*<0.001) and MWM (*P*<0.001). Concurrent administration of glycine (Group 2) provided the greatest protection, resulting in no significant decline in the Y-maze (*P* = 0.481) or MWM (*P* = 0.986). ANOVA confirmed highly significant overall differences in performance across groups for both the Y-Maze (*P*<0.001, *F*(4,20) = 108.995) and MWM (*F* = 52.878, *P*<0.0001). Delayed glycine also showed mitigation, with Group 5 performing significantly better than Group 3 in the MWM (*P* = 0.003).

**Conclusion:**

Glycine co-administration largely prevents spatial learning and working memory deficits caused by cisplatin in mice. These findings support the view that cisplatin impairs cognition through inflammatory and oxidative pathways, and suggest that targeting these pathways with simple interventions can be effective. Glycine, an inexpensive and safe naturally occurring molecule, holds promise for mitigating chemotherapy-related cognitive decline during cancer treatment.

## Introduction

Cisplatin, cis-diamminedichloroplatinum II, is one of the most widely used platinum-based chemotherapeutic agents, and it remains a cornerstone treatment for multiple solid tumors including testicular, ovarian, head and neck, lung, and bladder cancers. Globally, the cancer burden is large and growing, with about 20 million new cancer cases and nearly 9.7 million cancer deaths in 2022, and an increasing number of cancer survivors who may experience treatment-related late effects ^[^1,2^]^.

HIGHLIGHTS

- Glycine co-administration prevents cisplatin-induced cognitive decline in mice.
- Mice treated with glycine concurrently with cisplatin showed no significant cognitive impairment.
- Delayed glycine treatment partially mitigated cognitive decline.
- Glycine’s neuroprotective effects are likely due to its antioxidant and anti-inflammatory properties.

Despite its efficacy, cisplatin causes substantial toxicity that limits dosing and impacts quality of life. Major treatment-limiting toxicities include nephrotoxicity, peripheral neuropathy, severe nausea and vomiting, ototoxicity, and myelosuppression. These adverse effects are well documented in prescribing information and systematic reviews, and they contribute both to acute morbidity during therapy and to chronic functional impairments in survivors^[^3,4^]^.

Among the central nervous system consequences of chemotherapy, cognitive impairment, often referred to as “chemo-brain” or chemotherapy-related cognitive impairment, is increasingly recognized as an important survivorship issue. Meta-analyses and systematic reviews estimate that clinically relevant cognitive deficits affect roughly one in three cancer survivors in many cohorts, with impairments in attention, working memory, processing speed, and executive function reported across multiple cancer types and chemotherapy regimens. These deficits can persist months to years after treatment, reducing return to work, daily functioning, and overall quality of life^[^5,6^]^.

Preclinical and clinical evidence indicate that cisplatin can cross the blood–brain barrier and accumulates in brain regions critical for learning and memory, particularly the hippocampus and prefrontal cortex. In experimental models, cisplatin exposure produces oxidative stress, mitochondrial dysfunction, DNA damage, synaptic loss, and activation of neuroinflammatory signaling, including NF-κB activation and elevated cytokines such as TNF-α, IL-1β, and IL-6. These molecular and cellular changes translate into impaired synaptic plasticity, reduced neurogenesis, and deficits in spatial learning and memory on standard behavioral tests^[^7,8^]^.

The public health impact of chemotherapy-related cognitive impairment is magnified by the growing population of cancer survivors. With surviving cohorts expanding worldwide, particularly in low- and middle-income regions where cancer incidence is rising, interventions that prevent or mitigate cognitive late effects could have substantial population-level benefits. Early, affordable, and safe therapeutic strategies are therefore highly desirable^[^9,10^]^.

Glycine is the simplest amino acid, and it performs multiple roles relevant to neuroprotection. It acts as an inhibitory neurotransmitter in the spinal cord and brainstem, serves as a co-agonist at the NMDA receptor influencing synaptic plasticity, and contributes to glutathione synthesis, thereby supporting antioxidant defenses^[^11–14^]^. Mechanistically, glycine exerts anti-inflammatory effects by inhibiting NF-κB signaling and by reducing Ca^2^⁺-dependent cytokine release from immune cells, while maintaining cellular redox homeostasis through glutathione synthesis pathways^[^15,16^]^. Experimental studies across models of ischemia, aging, and toxic injury have shown that glycine supplementation reduces pro-inflammatory cytokines such as TNF-α and IL-1β, preserves neuronal morphology, and improves behavioral performance in learning and memory paradigms[17].

Taken together, the pathophysiology of cisplatin-induced cognitive dysfunction overlaps strongly with processes modulated by glycine namely oxidative stress, NF-κB driven neuroinflammation, and impaired synaptic transmission. This mechanistic convergence provides a strong rationale to test whether glycine can prevent or reverse cisplatin-induced cognitive deficits.

Animal models, particularly murine (mouse) models, offer an ethically and scientifically valid platform for this investigation[18]. Mice possess well-characterized neuroanatomy and neurochemistry that closely mirror key aspects of the human brain, including hippocampal structure, synaptic organization, and N-methyl-D-aspartate (NMDA) receptor physiology^[^19–22^]^. Their cognitive behavior can be quantitatively assessed through standardized tests such as the Morris water maze (MWM) and Y-maze, which reliably measure spatial learning and working memory^[^23–26^]^. Furthermore, cisplatin administration in mice recapitulates both the biochemical and behavioral hallmarks of human chemotherapy-induced cognitive impairment, i.e., oxidative stress, mitochondrial dysfunction, and cytokine driven neuroinflammation, allowing for mechanistic insights that cannot be ethically or practically obtained in humans at this stage[27]. Conducting early-phase preclinical research in mice thus provides essential proof-of-concept data on efficacy and safety before translation into human clinical studies.

The present study therefore evaluates the neuroprotective effects of glycine against cisplatin-induced cognitive decline in mice, using the MWM and Y-maze behavioral paradigms as primary functional endpoints. We hypothesize that glycine administration will ameliorate cisplatin-associated deficits in spatial learning and working memory and that behavioral protection will align with reduced inflammatory signaling and preserved neuronal integrity.

This study was conducted without the use of artificial intelligence tools in data collection and analysis, in compliance with the TITAN Guidelines 2025[28].

## Materials and methods

All experimental procedures and reporting in this study were conducted in accordance with the ARRIVE (Animal Research: Reporting of In Vivo Experiments) guidelines to ensure methodological rigor, transparency, and reproducibility[29].

### Animals

Twenty-five healthy adult male BALB/c mice, 6–8 weeks of age and weighing 22–28 g, were used in this study[30]. Young adult mice were selected because this age range exhibits stable behavioral and physiological responses, reducing variability in cognitive and pharmacological outcomes. BALB/c mice were selected due to their well-established behavioral and physiological responses, particularly in neurobiological research. Female mice and male diseased mice were excluded because the hormonal cycle variations in estrous could influence neuroinflammation and cognitive results. BALB/c mice were chosen owing to their well-characterized immunological and neurological systems, which make them particularly suitable for neurobiological and behavioral investigations[31]. Prior to experimentation, animals were acclimatized for 7 days to the laboratory environment under standard conditions (temperature 22 ± 2°C, humidity 50–60%, 12-hour light/dark cycle). Mice were housed in standard polypropylene cages, with a maximum of five animals per cage, and were provided standard laboratory chow and water ad libitum. Environmental enrichment, including cardboard tubes and nesting material, was supplied to encourage natural behaviors and reduce anxiety.

### Ethics considerations

All procedures strictly followed national and international ethical standards for animal research, including the Animal Welfare Act, the Public Health Service Policy, and the principles of the three Rs (Replacement, Reduction, and Refinement)^[^32–34^]^. Experimental protocols were approved by the Khyber Medical University Advance Studies & Research Board (Approval No. DIRJKMU-AS&RB/GA/002952, dated 16-10-2024). Humane endpoints, proper anesthesia, and regular behavioral and health monitoring were implemented to minimize animal distress throughout the study.

### Experimental design, group allocation, and sample size

This was conducted over a period of 6 months at Institute of Basic Medical sciences (IBMS) Khyber Medical University of Peshawar. For this study, the sample size was determined using the resource equation method[35]:

df = 20 → n = degrees of freedom (df)/k + 1 = 20/5 + 1 = 5 mice per group.

The mice were randomly assigned to one of five distinct experimental groups. This 28-day treatment program was designed to assess the neuroprotective capabilities of glycine when administered at varying times relative to cisplatin exposure:

1. Group 1 received only cisplatin for 14 days (assessing short-term toxicity).

2. Group 2 received cisplatin + glycine concurrently for 14 days (testing protective mechanisms).

3. Group 3 received only cisplatin for 28 days (examining long-term toxicity without intervention).

4. Group 4 received cisplatin for 14 days, followed by glycine for 14 days.

5. Group 5 received cisplatin for 28 days, with glycine administered from day 14 to day 28.

This design permitted researchers to thoroughly examine the preventive effects of glycine (Group 2), as well as its potential to act as a therapeutic agent through delayed administration (Groups 4 and 5), against the cognitive induced by cisplatin exposure. These groups are well visualized in Table 1.

**Table 1:** Experimental groups.

Group	Treatment protocol	Duration and intervention
Group 1	Cisplatin only	Cisplatin for 14 days
Group 2	Cisplatin + glycine (concurrent)	Cisplatin and glycine concurrently for 14 days
Group 3	Cisplatin only	Cisplatin for 28 days
Group 4	Cisplatin followed by glycine (reversal)	Cisplatin for 14 days, followed by glycine for the next 14 days
Group 5	Cisplatin + delayed glycine	Cisplatin for 28 days, with glycine administered from day 14 to day 28

### Randomization and blinding

The experimental design utilized strict procedures for subject allocation to ensure an unbiased distribution of the mice across the treatment cohorts. The study employed a simple random sampling technique for animal selection and grouping. This randomization was essential to minimize selection bias and ensure that the effects observed were attributable solely to the administered treatments.

### Treatment administration

The treatment phase of the study involved the precise administration of both cisplatin and glycine to the adult male BALB/c mice according to the designated experimental group protocols.

### Cisplatin administration

Cisplatin was administered at a dose of 3 mg/kg3[36]. The drug was delivered intraperitoneally (I.P.) every fourth day for the designated duration of the treatment plan. It was diluted using sterile saline into suitable doses tailored to match the individual mouse body weights. The resulting volume for I.P. administration ranged from 0.1 to 0.2 ml. A sterile insulin syringe (typically 1 ml with 29–31 gauge) was used for injection. Researchers held the mice gently and executed shallow angle needle entry into the lower right abdominal area. Proper sterile aseptic procedures were maintained to prevent infections and tissue damage. This administration schedule was intended to achieve a cumulative neurotoxic dosage of 11–14 mg by the end of the treatment period[36]. For groups receiving 14 days of cisplatin, the total cumulative dose was (4 doses of). No anesthetic was reported for injections.

### Glycine administration

Glycine was given at a dose of 1 g/kg/day[17]. It was administered subcutaneously (S.C.) every day[17]. In groups where glycine was administered alongside cisplatin (Group 2 and parts of Group 5), it was given 15 minutes before injecting cisplatin. The solution was prepared from powdered glycine using diluted distilled water. Glycine solution was injected into the dorsal scruff of the mice using a 1-ml insulin syringe with a 29–31 gauge. The administered volume (ranging from 0.1 to 0.2 ml) was calculated based on the mouse’s body weight to ensure accuracy. The S.C. route was chosen because it offered reliable drug absorption and allowed for easy repetition throughout the research period. No anesthetic was reported for injections.

### Behavioral assessments

The assessment of cognitive function was carried out for evaluating the neuroprotective effects of glycine and was conducted both before and after the 28 day treatment regimen using two standardized behavioral assays: the Y-maze and the MWM tests.

The Y-maze test was employed to evaluate spatial working memory and cognitive flexibility[37]. The apparatus comprised a Y-shaped structure made of three arms (30 × 15 × 15 cm). Each mouse was placed in the center of the maze and permitted free exploration for 8 minutes. Cognitive ability was quantified by calculating the spontaneous alternation percentage (SAP), determined by dividing the number of successful entries into three different arms by the total number of arm entries minus two, then multiplied by 100. A greater percentage of alternation behavior showed enhanced spatial working memory.

The MWM test was utilized to assess spatial learning and memory The apparatus consisted of a circular water tank filled with colored water, with the temperature maintained at 25^O^C^[^26,38^]^.

The test was composed of three phases: an acclimatization phase (Days 1–3) with a visible platform; a training phase (Days 4–7) involving four daily trials where mice located a hidden, submerged platform using spatial cues; and a testing phase (Day 8), or probe test, where the platform was removed.

During the testing phase, the key metric was the latency to reach the platform. Performance data, including latency and target quadrant duration, were objectively measured and recorded using a video tracking system. Overall, the mice were tested to find their way to a submerged platform in a pool of water in order to evaluate their spatial learning, memory, and latency to reach the platform.

### Statistical analysis

The statistical evaluation of the data was conducted using established scientific software and methods.

#### Software and presentation

Data analysis was carried out using SPSS (Statistical Package for the Social Sciences) software, specifically version 26, USA[39]. Quantitative variables collected throughout the study, including metrics from behavioral assessments, are presented in the results as mean ± standard deviation. The statistical significance level was reported as *P*<0.05 (also referred to as the threshold for clarity), and data were presented as mean ± SEM (standard error of the mean) in the methods section and results summary. Results were visually presented through figures, charts, and tables generated using SPSS4[39].

Primary statistical tests:

1. Paired T-tests: These were specifically applied to assess within-group differences for the behavioral measures. This test compared the performance of mice in the Y-maze and the MWM tests before and after the administration of treatments

2. One-way ANOVA (analysis of variance): This test was used to analyze group comparisons for the overall results of the Y-maze and MWM tests. Highly significant differences were confirmed among the treatment groups for Y-Maze (*P*<0.001, *F*(4,20) = 108.995) and MWM (*F* = 52.878, *P*<0.0001).

3. Tukey post hoc tests: These tests were executed following a significant result from the ANOVA. It allowed to pinpoint the specific individual group comparisons that exhibited significant differences, which was crucial for comprehending how different glycine treatments affected cognitive performance.

## Results

### Y-maze test

The Y-maze test was performed to assess spatial working memory and cognitive flexibility in the mice[37]. This assessment measured the sequence of arm entries (time spent in the novel and familiar arms of the maze) and calculated the SAP, with a greater percentage of alternation behavior signifying enhanced spatial working memory. Statistical analysis using one-way ANOVA confirmed highly significant differences in cognitive performance across the five treatment groups (*P*<0,001, *F*(4,20) = 108.995).

#### Effects of cisplatin administration

Paired samples t-tests comparing pre- and post-treatment performance revealed that mice receiving cisplatin alone experienced significant cognitive decline. Specifically, Group 1 (cisplatin for 14 days) and Group 3 (cisplatin for 28 days) both showed a substantial reduction in time spent in the preferred arm following treatment (*P* = 0.000 for both groups). This outcome demonstrates the neurotoxicity induced by cisplatin, leading to impaired cognitive function (refer to Table 2 and Table 3 for Y-maze test statistics).

**Table 2:** Time spent in the maze arms for each treatment group.

Y-maze test paired samples statistics	Mean	*N*	Std. deviation	Std. error mean	*P*-value
Pair 1 Group 1 pre-treatment	75.4000	5	2.30217	1.02956	0.000
Group 1 post-treatment	42.4000	5	5.02991	2.24944	
Pair 2 Group 2 pre-treatment	77.8000	5	1.92354	0.86023	0.481
Group 2 post-treatment	79.4000	5	4.15933	1.86011	
Pair 3 Group 3 pre-treatment	73.2000	5	1.92354	0.86023	0.000
Group 3 post-treatment	32.6000	5	3.57771	1.60000	
Pair 4 Group 4 pre-treatment	76.2000	5	1.92354	0.86023	0.011
Group 4 post-treatment	68.6000	5	3.64692	1.63095	
Pair 5 Group 5 pre-treatment	75.4000	5	2.07364	0.92736	0.0078
Group 5 post-treatment	62.2000	5	3.96232	1.77200

**Table 3:** Comparison between the groups with reference to Y-maze test.

ANOVA	Sum of squares	df	Mean square	*F*	Significance
Between groups	7359.360	4	1839.840	108.995	0.000
Within groups	337.600	20	16.880		
Total	7696.960	24			

#### Neuroprotective effects of glycine

Glycine administration significantly mitigated these deficits, demonstrating its neuroprotective potential.

##### Concurrent administration (Group 2)

Mice receiving glycine alongside cisplatin for 14 days (Group 2) exhibited no significant decline in cognitive function post-treatment, with a paired samples *P*-value of 0.481. This result suggests that concurrent glycine effectively prevents cisplatin-induced cognitive impairment.

##### Delayed administration (Groups 4 and 5)

Groups where glycine treatment followed or occurred during ongoing cisplatin exposure showed a lesser degree of cognitive impairment compared to cisplatin-only groups. Group 4 (cisplatin for 14 days followed by glycine for 14 days) reported a *P*-value of 0.011, and Group 5 (cisplatin for 28 days with glycine from day 15) reported a *P*-value of 0.007.

The overall findings demonstrated that the effects of glycine were more pronounced when administered concurrently but were still evident with delayed administration.

#### Post hoc analysis

**Figure 1.**
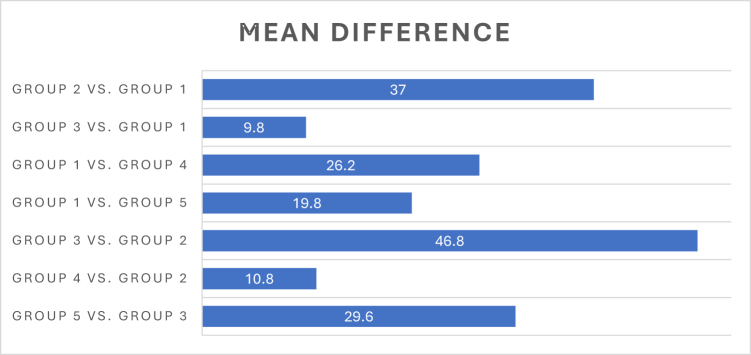
Bar showing comparison between the groups for Y-maze test.

Post hoc comparison tests further elucidated the significant differences between groups. Group 2 (cisplatin + glycine) spent significantly more time in the novel arm compared to Group 1 (cisplatin only), with a highly significant mean difference (37.00) and *P* = 0.000. The lowest cognitive performance, indicated by the least time spent in the novel arm, was observed in Group 3 (Cisplatin 28 days). Groups 4 and 5 also showed improved performance when compared to cisplatin-only groups, indicating the potential benefits of glycine treatment in partially restoring cognitive abilities impaired by cisplatin. The post hoc comparison statistics for the Y-maze test are presented in Table 4. The main differences are plotted in Figure 1.

**Table 4:** Comparison between the groups with reference to Y-maze test.

Post hoc tests	Comparison	Mean difference	Std. error	Significance^*^	95% Confidence interval (lower bound–upper bound)
	Group 2 vs Group 1	37	2.59846	0	29.2244–44.7756
	Group 3 vs Group 1	9.8	2.59846	0.009	2.0244–17.5756
	Group 1 vs Group 4	26.2	2.59846	0	18.4244–33.9756
	Group 1 vs Group 5	19.8	2.59846	0	12.0244–27.5756
	Group 3 vs Group 2	46.8	2.59846	0	39.0244–54.5756
	Group 4 vs Group 2	10.8	2.59846	0.004	3.0244–18.5756
	Group 5 vs Group 3	29.6	2.59846	0	21.8244–37.3756

^*^ The mean difference is significant at the 0.05 level

Overall reinforcing that the greatest cognitive protection was observed when glycine was administered concurrently with cisplatin, while delayed administration partially restored cognitive abilities impaired by cisplatin.

### MWM test

The MWM test was utilized to evaluate spatial learning and memory function by measuring the time (latency) required for mice to locate a hidden platform following the 28-day treatment regimen. Analysis of the post-treatment latency revealed highly significant differences in cognitive outcomes among the five groups, as confirmed by one-way ANOVA (*F* = 52.878, *P*<0.0001). Refer to Table 5.

**Table 5:** Comparison between the groups with reference to Morris water maze test.

ANOVA	Sum of squares	df	Mean square	*F*	Significance
Between groups	20 629.307	4	5157.327	52.878	0.000
Within groups	1950.643	20	97.532		
Total	22 579.950	24			

#### Impact of cisplatin on spatial memory

Mice subjected to cisplatin alone demonstrated substantial cognitive impairment. Paired samples t-tests comparing pre- and post-treatment latencies showed a significant increase in the time taken to reach the platform for Group 1 (cisplatin 14 days) (*P* = 0.000) and Group 3 (cisplatin 28 days) (*P* = 0.001). The effects were particularly pronounced in Group 3, consistent with the severe neurotoxicity resulting from prolonged cisplatin exposure. Refer to Table 6.

**Table 6:** Time taken to reach the platform for each treatment group.

Morris water test paired samples statistics	Mean	*N*	Std. deviation	Std. error mean	*P*-value
Pair 1 Group 1 pre-treatment	68.2400	5	4.34276	1.94214	0.000
Group 1 post-treatment	102.1240	5	6.40616	2.86492	
Pair 2 Group 2 pre-treatment	51.3000	5	2.14476	0.95917	0.986
Group 2 post-treatment	51.3580	5	6.16620	2.75761	
Pair 3 Group 3 pre-treatment	82.3060	5	3.10167	1.38711	0.001
Group 3 post-treatment	139.5300	5	16.40417	7.33617	
Pair 4 Group 4 pre-treatment	71.2720	5	2.62751	1.17506	0.000
Group 4 post-treatment	108.2620	5	9.32678	4.17106	
Pair 5 Group 5 pre-treatment	90.1020	5	3.75119	1.67758	0.006
Group 5 post-treatment	112.8100	5	7.24666	3.24081	

#### Neuroprotective and mitigation effects of glycine

Glycine administration effectively mitigated these deficits:

##### Concurrent administration (Group 2)

Mice receiving glycine alongside cisplatin for 14 days (Group 2) showed no significant change in post-treatment latency (*P* = 0.986). This strongly suggests that concurrent glycine effectively preserved spatial learning and memory function. Group 2 demonstrated the shortest time to reach the platform among all groups, reflecting enhanced learning and memory capabilities.

##### Delayed administration (Groups 4 and 5)

Groups receiving delayed glycine (Groups 4 and 5) also showed significant increases in post-treatment latency (*P* = 0.000 and *P* = 0.006, respectively); however, the overall cognitive decline was less severe compared to mice treated with cisplatin alone.

#### Post hoc analysis of group differences

**Figure 2.**
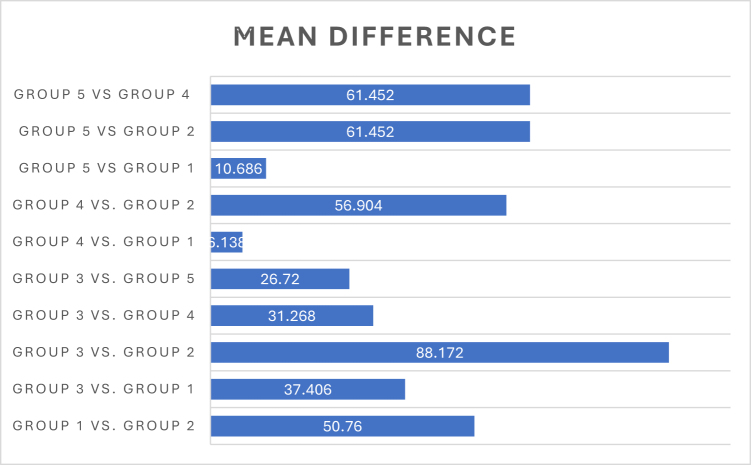
Bar showing comparison between the groups for Morris water maze test.

Post hoc analyses using Tukey’s test confirmed the severity of impairment and the efficacy of glycine. Group 3 exhibited significantly worse performance across multiple comparisons, particularly against Group 2, with an overall mean difference of 88.172 (*P* = 0.000). Group 2 showed highly significant protective effects when compared to Group 1 (mean difference 50.766, *P* = 0.000). While delayed administration showed benefits, the difference between Group 4 and Group 1 was not statistically significant (mean difference 6.138, *P* = 0.86), suggesting concurrent prevention offers superior protection, though Group 5 showed significantly better results than Group 3 (mean difference 26.72, *P* = 0.003), supporting its mitigative potential when introduced later. Refer to Table 7 and Figure 2.

**Table 7:** Comparison between the groups with reference to Morris water maze test.

Post hoc tests (mean differences between groups)	Comparison	Mean difference	Std. error	Significance^*^	95% Confidence interval
	Group 1 vs Group 2	50.766	6.24603	0	32.0755–69.4565
	Group 3 vs Group 1	37.406	6.24603	0	18.7155–56.0965
	Group 3 vs Group 2	88.172	6.24603	0	69.4815–106.8625
	Group 3 vs Group 4	31.268	6.24603	0.001	12.5775–49.9585
	Group 3 vs Group 5	26.72	6.24603	0.003	8.0295–45.4105
	Group 4 vs Group 1	6.138	6.24603	0.86	−12.5525–24.8285
	Group 4 vs Group 2	56.904	6.24603	0	38.2135–75.5945
	Group 5 vs Group 2	61.452	6.24603	0	42.7615–80.1425
	Group 5 vs Group 4	61.452	6.24603	0.947	−14.1425 − 23.2385
	Group 5 vs Group 1	10.686	6.24603	0.45	−8.0045–29.3765

^*^ The mean difference is significant at the 0.05 level

## Discussion

This study tested whether glycine, a simple amino acid with known antioxidant and NMDA receptor-modulating properties could prevent or reverse the spatial learning and memory deficits caused by cisplatin chemotherapy in mice. Using standard behavioral assays, we found that cisplatin alone produced marked cognitive impairments: mice treated with cisplatin (Groups 1 and 3) showed significantly longer escape latencies in the MWM and reduced alternation behavior in the Y-maze, consistent with the “chemo-brain” deficits reported in other models^[^7,40^]^. In contrast, mice that received glycine concurrently with cisplatin (Group 2) performed comparably to controls, exhibiting no significant post-treatment decline in either task (paired-test *P* ≈ 0.48 for Y-maze; *P* ≈ 0.99 for MWM). Even delayed glycine treatment (Groups 4–5) yielded partial rescue: these mice showed less severe deficits than cisplatin-only controls, though still worse performance than the concurrent-glycine group. In sum, our primary outcomes indicate that glycine robustly protected mice from cisplatin-induced working and spatial memory loss when given prophylactically, and offered some mitigation even when administered after cisplatin exposure. Notably, the greatest protection was seen with concurrent glycine, suggesting a preventive mechanism. These findings establish glycine as an effective neuroprotectant in this model.

Our results are in broad agreement with prior preclinical studies of “chemo brain.” For example, Zhou and colleagues showed that cisplatin causes clear cognitive deficits in mice impairing performance in novel object recognition and other memory tasks and that co-treatment with protective agents (metformin) prevented these deficits[41]. Similarly, Aldubayan *et al* recently demonstrated that cisplatin impairs spatial learning and working memory in rats via hippocampal inflammation and apoptosis and that co-treatment with an oat-derived anti-inflammatory compound (avenanthramide C) reversed these effects[42]. In the same vein, edaravone (a free radical scavenger) and N-acetylcysteine (NAC) have been reported to reduce cisplatin-related hippocampal oxidative stress and improve memory scores[27]. Our findings with glycine parallel these reports: all suggest that anti-inflammatory/antioxidant interventions can counteract cisplatin neurotoxicity. Importantly, no previous study (to our knowledge) has tested glycine in this context, but the current results align with glycine’s known neuroprotective effects in other models[42]. Human data likewise indicate that chemotherapy often causes lasting cognitive impairment estimated at roughly one-third of survivors in meta-analyses, so our model appears to capture a relevant aspect of patient experience^[^5,43^]^. In summary, the consistency of our data with the broader literature (including systemic reviews of animal models[40]) supports the validity of the mouse behavioral changes we observed.

Several systematic reviews and meta-analyses reinforce our interpretation. A comprehensive review of preclinical studies found that cisplatin, as well as other common chemotherapies, reliably produces deficits in short and long-term memory in rodents[40]. These impairments were consistently linked to hippocampal pathology, e.g., reduced neurogenesis, increased inflammation and mitochondrial damage, in line with our expectation^[^7,40^]^. Likewise, patient focused meta-analyses report that roughly 20–30% of chemotherapy treated survivors show neuropsychological deficits by standardized testing^[^5,43^]^. Our work fits with this consensus: glycine reversed dysfunction in precisely those domains (spatial and working memory) that are among the most vulnerable in chemo-brain. To our knowledge, no meta-analysis has specifically examined glycine supplementation in chemo patients, but glycine has been reviewed for other conditions (e.g. aging related impairment) with generally positive effects on cognitive outcomes. Thus, our results do not contradict any existing systematic findings. If anything, they suggest a new potential therapy worth aggregating in future reviews of CRCI interventions.

Biologically, our data are consistent with known pathways of cisplatin neurotoxicity and glycine action. Cisplatin is known to induce oxidative stress and pro-inflammatory cytokine release in the brain^[^7,42^]^. In our study, the exacerbated latencies and reduced alternation behavior after cisplatin are plausibly due to hippocampal injury: for example, recent work showed cisplatin markedly increases hippocampal NF-κB, TNF-α, IL-6, and reactive oxygen species in rats, which correlates with impaired working memory on Y-maze tasks[7]. Glycine may counter these insults via multiple mechanisms. It is a precursor for glutathione, so glycine supplementation can bolster antioxidant defenses. Glycine also acts as a co-agonist at NMDA receptors, which could stabilize synaptic transmission during stress. Intriguingly, cisplatin was reported to upregulate AMPA and NMDA receptor expression, potentially causing excitotoxicity[7]. By providing glycine at the NMDA site, one might modulate receptor activity to reduce excitotoxic signaling. Additionally, glycine has known anti-inflammatory effects: it can inhibit NF-κB signaling and lower TNF-α/IL-1β release in the CNS. Indeed, glycine treatment in models of neurodegeneration suppressed gliosis and cytokine production. Thus, the preserved memory in our glycine treated mice likely reflects a combination of antioxidant and anti-inflammatory neuroprotection, as well as maintained synaptic plasticity (via NMDA site modulation). Our study did not measure cytokines or oxidative markers directly, so this mechanistic interpretation is necessarily inferential, but it is strongly supported by the literature[7].

This study has several strengths. It used a controlled experimental design with randomization and blinding and included both acute and chronic cisplatin regimens with multiple glycine timing conditions. The use of two distinct behavioral assays (MWM and Y-maze) strengthens internal validity, as consistent effects across tasks reduce the likelihood of a task-specific artifact. Group sizes were chosen by power calculations (*n* =5 each), and statistical analyses (ANOVA with post hoc tests) were robust to confirm group differences.

## Limitations

The sample size is modest by human-trial standards, and we used only male BALB/c mice; effects in females or other strains are unknown. We did not include a glycine-only control group, though glycine alone is not expected to impair cognition. Glycine is generally regarded as safe even at high doses in rodents. Our behavioral measures were taken shortly after treatment; longer-term follow up would be needed to see if glycine’s benefit persists. Longitudinal studies are needed to determine whether glycine confers transient relief or lasting neuroprotection.

Finally, like most animal studies, translation to humans is uncertain: the cisplatin dose and schedule differ from clinical regimens, and it is unclear whether oral glycine (the human-relevant route) would achieve similar brain levels. Thus, external validity is limited, and future work must address these gaps.

## Recommendations and future directions

We recommend testing glycine in tumor-bearing models to ensure that it does not protect cancer cells or interfere with chemotherapy efficacy. Dose–response studies are needed: we used one glycine dose (injected), but it will be important to define an optimal regimen and to test oral administration.

Mechanistic studies should accompany behavior, i.e., markers of oxidative damage in treated mice could confirm glycine’s anti-inflammatory effect. Investigating synaptic markers (e.g., PSD95, synaptophysin) would test whether glycine preserved synaptic integrity. It may also be worthwhile to compare glycine with other NMDA modulators or antioxidants in head-to-head studies. Finally, if glycine continues to show promise, early translational work could explore its use in cancer survivors. Because glycine is inexpensive and generally safe, clinical trials could be designed (e.g., adjunctive glycine in patients on cisplatin) once basic safety and dose data are established.

## Conclusion

This study demonstrates that glycine co-administration largely prevents the spatial learning and working memory deficits caused by cisplatin in mice. These results reinforce the view that cisplatin impairs cognition through inflammatory and oxidative pathways, and suggest that targeting these pathways with simple interventions can be effective. By linking glycine’s effects to known neuroprotective mechanisms, this work adds to the growing body of literature on chemo-brain and its prevention. Overall, the main insight is that glycine, a safe, naturally occurring molecule, holds promise for mitigating chemotherapy-related cognitive decline, during cancer treatment.

## Data Availability

All data supporting the findings of this study are included within the main document.

## References

[1] World Health Organization: WHO. Cancer. Published February 3, 2025. https://www.who.int/news-room/fact-sheets/detail/cancer

[2] Bray F Laversanne M Sung H. Global cancer statistics 2022: GLOBOCAN estimates of incidence and mortality worldwide for 36 cancers in 185 countries. CA a Cancer J Clin 2024;74:229–63. 10.3322/caac.21834 38572751

[3] Gold JM Raja A. Cisplatin. StatPearls - NCBI Bookshelf. Published May 22, 2023. https://www.ncbi.nlm.nih.gov/books/NBK547695/

[4] Callejo A Sedó-Cabezón L Juan I. Cisplatin-induced ototoxicity: effects, mechanisms and protection strategies. Toxics 2015;3:268–93. 29051464 10.3390/toxics3030268 PMC5606684

[5] Whittaker AL George RP., and O’Malley L. Prevalence of cognitive impairment following chemotherapy treatment for breast cancer: a systematic review and meta-analysis. Sci Rep 2022;12:2135. 10.1038/s41598-022-05682-1 PMC8826852 35136066

[6] Amani O Mazaheri MA Moghani MM. Chemotherapy-induced cognitive impairment in breast cancer survivors: a systematic review of studies from 2000 to 2021. Cancer Rep 2024;7:e1989. 10.1002/cnr2.1989 PMC10864736 38351543

[7] Alhowail AH. Cisplatin induces hippocampal neurotoxicity and cognitive impairment in rats through neuroinflammation, oxidative stress, and overexpression of glutamatergic receptors mRNA. Front Pharmacol 2025;16:1592511. 10.3389/fphar.2025.1592511 PMC12163336 40520187

[8] Santos NAGD Ferreira RS Santos ACD. Overview of cisplatin-induced neurotoxicity and ototoxicity, and the protective agents. Food Chem Toxicol 2019;136:111079. 31891754 10.1016/j.fct.2019.111079

[9] World Health Organization: WHO. Global cancer burden growing, amidst mounting need for services. World Health Organization. https://www.who.int/news/item/01-02-2024-global-cancer-burden-growing–amidst-mounting-need-for-services. Published February 1, 2024.

[10] Global Cancer Facts & Figures. American Cancer Society. Accessed 20 November 2024. https://www.cancer.org/research/cancer-facts-statistics/global-cancer-facts-and-figures.html

[11] Béchade C Sur C Triller A. The inhibitory neuronal glycine receptor. BioEssays 1994;16:735–44. 7980477 10.1002/bies.950161008

[12] Li Y Krupa B Kang JS. Glycine site of NMDA receptor serves as a spatiotemporal detector of synaptic activity patterns. J Neurophysiol 2009;102:578–89. 19439669 10.1152/jn.91342.2008 PMC2712282

[13] McCarty MF O’Keefe JH DiNicolantonio JJ. Dietary glycine is rate-limiting for glutathione synthesis and may have broad potential for health protection. Ochsner J 2018;18:81–87. 29559876 PMC5855430

[14] Forman HJ Zhang H Rinna A. Glutathione: overview of its protective roles, measurement, and biosynthesis. Mol Aspects Med 2008;30:1–12. 18796312 10.1016/j.mam.2008.08.006 PMC2696075

[15] Aguayo-Cerón KA Sánchez-Muñoz F Gutierrez-Rojas RA. Glycine: the smallest anti-inflammatory micronutrient. Int J Mol Sci 2023;24:11236. 37510995 10.3390/ijms241411236 PMC10379184

[16] Blancas-Flores G Alarcón-Aguilar FJ García-Macedo R. Glycine suppresses TNF-α-induced activation of NF-κB in differentiated 3T3-L1 adipocytes. Eur J Pharmacol 2012;689:270–77. 22732655 10.1016/j.ejphar.2012.06.025

[17] Ullah R Jo MH Riaz M. Glycine, the smallest amino acid, confers neuroprotection against D-galactose-induced neurodegeneration and memory impairment by regulating c-Jun N-terminal kinase in the mouse brain. J Neuroinflammation 2020;17:303. 33059700 10.1186/s12974-020-01989-w PMC7566050

[18] Justice MJ Dhillon P. Using the mouse to model human disease: increasing validity and reproducibility. Dis Model Mech 2016;9:101–03. 26839397 10.1242/dmm.024547 PMC4770152

[19] Beauchamp A Yee Y Darwin BC. Whole-brain comparison of rodent and human brains using spatial transcriptomics. Elife 2022;11:e79418. 36342372 10.7554/eLife.79418 PMC9708081

[20] Ashbrook DG Williams RW Lu L. Joint genetic analysis of hippocampal size in mouse and human identifies a novel gene linked to neurodegenerative disease. BMC Genomics 2014;15:850. 25280473 10.1186/1471-2164-15-850 PMC4192369

[21] Bakken TE Jorstad NL Hu Q. Comparative cellular analysis of motor cortex in human, marmoset and mouse. Nature 2021;598:111–19. 34616062 10.1038/s41586-021-03465-8 PMC8494640

[22] Loomba S Straehle J Gangadharan V. Connectomic comparison of mouse and human cortex. Science 2022;377:eabo0924. 35737810 10.1126/science.abo0924

[23] Othman MZ Hassan Z Che Has AT. Morris water maze: a versatile and pertinent tool for assessing spatial learning and memory. Exp Anim 2022;71:264–80. 35314563 10.1538/expanim.21-0120 PMC9388345

[24] Prieur EAK Jadavji NM. Assessing spatial working memory using the spontaneous alternation Y-maze test in aged male mice. Bio Protoc 2019;9:e3162. 10.21769/BioProtoc.3162 PMC7854095 33654968

[25] Vorhees CV Williams MT. Assessing spatial learning and memory in rodents. ILAR J 2014;55:310–32. 25225309 10.1093/ilar/ilu013 PMC4240437

[26] Vorhees CV Williams MT. Morris water maze: procedures for assessing spatial and related forms of learning and memory. Nat Protoc 2006;1:848–58. 17406317 10.1038/nprot.2006.116 PMC2895266

[27] John J Kinra M Mudgal J. Animal models of chemotherapy-induced cognitive decline in preclinical drug development. Psychopharmacology (Berl) 2021;238:3025–53. 34643772 10.1007/s00213-021-05977-7 PMC8605973

[28] Riaz AA Ginimol M Rasha R. Transparency in the reporting of Artificial Intelligence – the TITAN guideline. Prem J Sci 2025;10:100082.

[29] Du Sert NP Ahluwalia A Alam S. Reporting animal research: explanation and elaboration for the ARRIVE guidelines 2.0. PLoS Biol 2020;18:e3000411. 32663221 10.1371/journal.pbio.3000411 PMC7360025

[30] Jackson SJ Andrews N Ball D. Does age matter? The impact of rodent age on study outcomes. Lab Anim 2016;51:160–69. 27307423 10.1177/0023677216653984 PMC5367550

[31] Sultana R Ogundele OM Lee CC. Contrasting characteristic behaviours among common laboratory mouse strains. R Soc Open Sci 2019;6:190574. 31312505 10.1098/rsos.190574 PMC6599779

[32] Animal welfare act | national agricultural library. Accessed 15 October 2025. https://www.nal.usda.gov/animal-health-and-welfare/animal-welfare-act

[33] PHS policy on humane care and use of laboratory animals | OLAW. Accessed 16 October 2025. https://olaw.nih.gov/policies-laws/phs-policy.htm

[34] UConn Health Russell W Jr., and Burch R Jr. The 3R’s: replacement, reduction, and refinement- an overview. 2016. Accessed 16 October 2025. https://ovpr.uchc.edu/wp-content/uploads/sites/2568/2015/08/The-ACC-Connection-104-FALL-2016.pdf

[35] Festing MFW Altman DG. Guidelines for the design and statistical analysis of experiments using laboratory animals. ILAR J 2002;43:244–58. 12391400 10.1093/ilar.43.4.244

[36] Perše M. Cisplatin mouse models: treatment, toxicity and translatability. Biomedicines 2021;9:1406. 34680523 10.3390/biomedicines9101406 PMC8533586

[37] Kraeuter AK Guest PC., and Sarnyai Z. The Y-Maze for assessment of spatial working and reference memory in mice. Meth Mol Biol 2019;105–11. doi:10.1007/978-1-4939-8994-2_10 30535688

[38] Morris R. Developments of a water-maze procedure for studying spatial learning in the rat. J Neurosci Methods 1984;11:47–60. 6471907 10.1016/0165-0270(84)90007-4

[39] IBM Corp. IBM SPSS Statistics for Windows, Version 26.0. Armonk, NY: IBM Corp; 2019.

[40] Matsos A Johnston IN. Chemotherapy-induced cognitive impairments: a systematic review of the animal literature. Neurosci Biobehav Rev 2019;102:382–99. 31063740 10.1016/j.neubiorev.2019.05.001

[41] Zhou W Kavelaars A Heijnen CJ. Metformin prevents cisplatin-induced cognitive impairment and brain damage in mice. PLoS ONE 2016;11:e0151890. 27018597 10.1371/journal.pone.0151890 PMC4809545

[42] Aldubayan MA. Avenanthramide C mitigates cisplatin-induced hippocampal neurotoxicity and cognitive impairment in rats via suppression of neuroinflammation and neuronal apoptosis. Front Pharmacol 2025;16. doi:10.3389/fphar.2025.1706224 PMC12620472 41256255

[43] He YQ Zhou CC Jiang SG. Natural products for the treatment of chemotherapy-related cognitive impairment and prospects of nose-to-brain drug delivery. Front Pharmacol 2024;15:1292807. 38348396 10.3389/fphar.2024.1292807 PMC10859466

